# Comparison of the finishing shot and ending zone of points in Grand Slam matches of women’s doubles tennis: A cross-sectional study

**DOI:** 10.1371/journal.pone.0303437

**Published:** 2024-05-21

**Authors:** Marcos Borderias, Xavier Iglesias, Rafael Martínez-Gallego, Ernest Baiget

**Affiliations:** 1 Institut Nacional d’Educació Física de Catalunya (INEFC), Universitat de Barcelona (UB), Barcelona, Spain; 2 Department of Sport and Physical Education, Universitat de València, Valencia, Spain; University of Mississippi, UNITED STATES

## Abstract

Despite the importance of doubles tennis, there is little research on this modality of the game, especially in women’s tennis. The aim of this study was to analyse the finishing shot, the ending zone and shot by ending zone of women´s doubles matches during three Grand Slams (GS), and to observe the differences among surfaces. Twenty-one women’s doubles matches from three GS, each played on a different surface were analysed. 56.4 ± 2.3% of the points were finished from the baseline and mid court zone (BMZ), 36.1 ± 2.3% from the net zone (NZ) and 7.5 ± 4.2% from the service zone (SZ). The shot with which the points ended primarily was the forehand volley (FV) (18.2 ± 3.5%), followed by the forehand (F) (17.4 ± 4.2%), the backhand (B) (15.8 ± 4.8%), the backhand volley (BV) (12.8 ± 3.6%), the forehand return (FRT) (11.7 ± 2.7%), the backhand return (BRT) (11.4 ± 2.6%), the service (S) (7.5 ± 4.2%) and the overhead (O) (5.2 ± 3.0%). The results showed a higher percentage of F played from the BMZ in Roland Garros (RG) than in Wimbledon (W) (p = 0.011). In addition, the results also showed a higher percentage in overall F in RG than in W (p = 0.023). The FRT percentage was higher in the Australian Open (AUS) compared to RG (p = 0.026), and the O shots played from the BMZ were higher in W than RG (p = 0.038). These results suggest that in professional women’s doubles tennis the net game is a determinant factor, as well as the volley (V) and F shots. Playing aggressive and close to the net may have a positive effect on the result.

## Introduction

Many professional tennis tournaments take place every year. These include tournaments by the Women’s Tennis Association (WTA), Association of Tennis Professionals (ATP), women’s and men’s International Tennis Federation (ITF), as well as women’s and men’s Grand Slams (GS) tournaments [1–3]. Doubles matches are played in all of these professional tournaments and doubles specialists and many singles players participate [4]. Professional tennis players’ participation in doubles matches is likely motivated by several factors. These include, but not limited to, the prize money being offered [5], the desire to improve various skills (service, return, reaction speed and perception) [6], and the preparation for team competitions such as the Davis Cup, Billie Jean King Cup, and ATP Cup, which place high importance on doubles tennis [5]. These professional tennis tournaments take place on different court surfaces throughout the year, including hard courts, clay courts, and grass courts. Performance characteristics of elite tennis match-play differ depending on the court surface, affecting the physical and physiological responses of players [7]. Given these differences across surfaces, it is important to analyse doubles matches played on all the various court types used in professional tournaments.

Certain aspects of the rules in doubles tennis differ from singles tennis, such as lines, spaces or scoring systems. Doubles is played in pairs, and the court dimensions are different, with added alleys on each side. Consequently, the precision and the direction of the shots is very important, as the space-to-player ratio is smaller. This affects the training, movements, positions, timing, and physiological demands compared to singles [5]. Doubles also involves greater tactical and decision-making complexity. With two players per team, there are more stimuli to process [6,8]. In terms of scoring format, doubles matches often utilize a best of two tiebreak set system, without advantage sets. However, GS doubles contests differ by playing best of three tiebreak sets with advantage sets, similar to singles [9,10]. These varied match formats impact total match duration. Additionally, communication between doubles partners is crucial. Verbal and non-verbal communication allows teams to effectively solve problems and make decisions during points [11]. In general, the numerous differences result in shorter match times compared to singles [5].

Despite the importance of doubles tennis, literature on the topic is limited. Existing research has examined elements such as doubles tactics [6,8,12,13], match structure and scoring [5], communication between partners [11,14], player coordination [15], the best doubles players [4], sex differences [16], scoring systems [17], time characteristics based on team experience [18], serve performance [19,20] and volley positions [21]. However, the majority of the aforementioned studies have focused exclusively on analyzing men’s doubles tennis performance. While some compare men’s and women’s doubles [6,8,16,20,21], none have centered solely on elite women’s doubles play. To date, only three studies have exclusively examined women’s professional doubles, investigating the team communication [11], activity profile [22] and game structure and point ending [23].

Regarding the match external load differences between men’s and women’s doubles at a professional level, if we observe the work-rest ratio of one study in women’s doubles [22] and another one in men’s doubles [18], the work-rest ratio seems slightly lower in the women’s one. Additionally, the total match time was slightly longer in women’s doubles matches played on hard and clay courts, but shorter on grass surfaces [18,22]. When examining differences between singles and doubles, it has been observed that rally duration is shorter in men’s doubles than singles [18]. However, the rally time in women’s doubles seems longer on the three surfaces compared to men’s singles and with no differences compared to women’s singles [23,24]. In terms of other timing variables, the work-rest ratio was lower in men’s and women’s doubles versus singles as well [18,22,24] and rest time between points and total rest time were higher in women’s doubles compared to singles [23]. Finally, looking at structural differences, it was noted that men’s doubles involved fewer sets, points per match, and points per game compared to women’s doubles, though more games were played per set [5,23].

To the authors’ knowledge, no research has analyzed the specific shots and court zones with which points end in either doubles or singles tennis matches. Thus, the aim of this study was to address this gap by describing the finishing shot, ending zone, and shot by ending zone in women’s doubles matches across three different GS tournaments. Differences across playing surfaces were also analyzed.

## Materials and methods

### Matches

The study was approved by the Clinical Research Ethics Committee of the Sports Administration of Catalonia, Spain (23/CEICGC/2020). The performance characteristics of 21 matches were analysed. The imaging recruitment period for this study began on March 3, 2020 and ended on April 24, 2020. According to the Ethical Principles of Psychologists and Code of Conduct of the American Psychological Association [25], given that the tennis matches were held in public settings and records of observation of public images were made, it was not necessary to request informed consent from the participants.

The videos were obtained from a public platform available to all GS players called Match Analysis Department. The matches were played at three GS of the 2019 season, which were played on three different surfaces: Australian Open (hard court, n = 7), Roland Garros (clay, n = 7) and Wimbledon (grass, n = 7). The observational record was made between April 28 and May 15, 2020. The study follows all the ethical guidelines of the Declaration of Helsinki. A total of 22 doubles pairs and 42 professional female players were observed (age 30.1 ± 5.6 years; weight 65.4 ± 6.5 kg; height 170.1 ± 27.8 cm), with a median WTA ranking of 24.5 (range: 1–481). Matches in the first round (n = 2), third round (n = 3), quarter-finals (n = 9), semi-finals (n = 4) and final (n = 3) were analysed.

### Procedures

**Fig 1:**
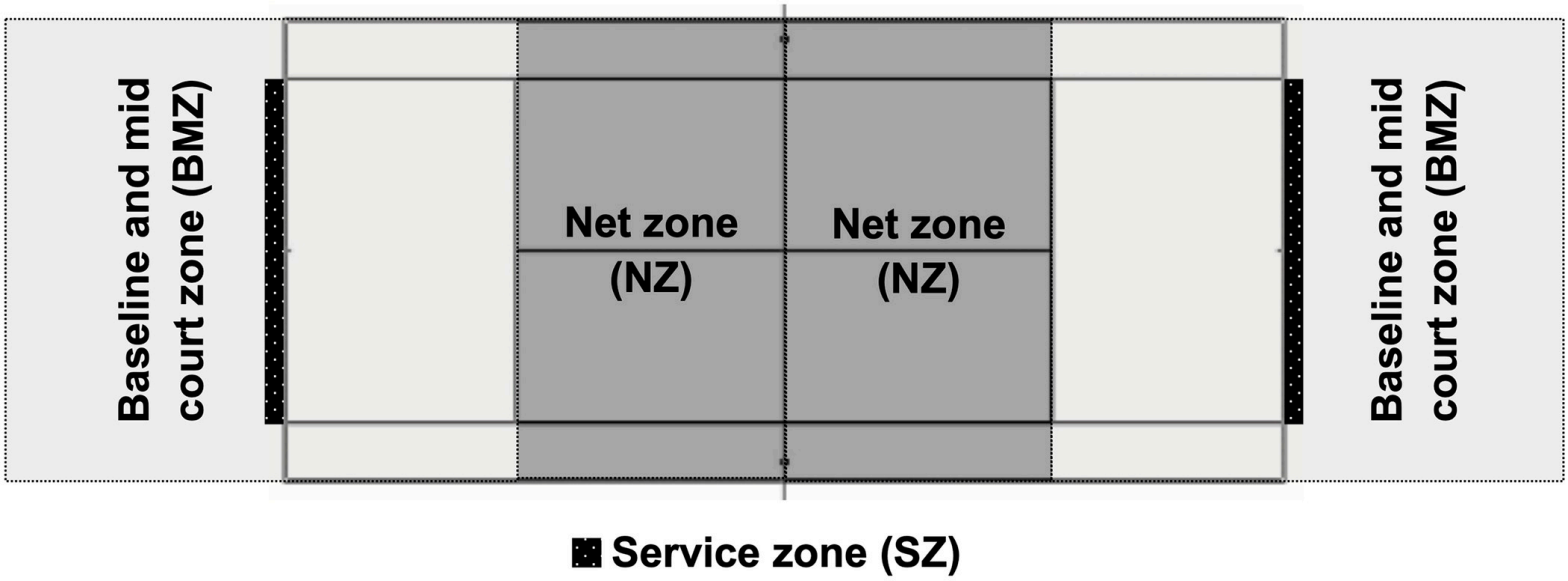
Ending zones. NZ, net zone; BMZ, baseline and mid court zone; SZ, service zone.

Online access to the footage of the tennis matches available on the web for GS players was obtained from a professional player. As we were part of the technical team of one of them, we could download the videos from the public platform for all the players and coaches. From these accounts the videos of the matches can be downloaded free of charge for their analysis in MP4 format with a frame size of 1280 x 720 pixels high and a frame rate of 25.00 frames per second. The videos did not need processing. For the observational recording, the LINCE PLUS v.1.4 [26] was used. An observational instrument was created *ad hoc* based on scientific literature related to the analysis of external load parameters in singles tennis [27–29], composed of 6 criteria and 18 categories collecting data on player, type of shot, outcome and location of the shots (Table 1). Points scored in each game, set and match were also recorded (Table 1) [22,23]. The analysis of the finishing shot, defined as the last shot played regardless of its outcome (winner, unforced error, forced error), included forehand (F), backhand (B), forehand volley (FV), backhand volley (BV) forehand return (FRT), backhand return (BRT), service (S) and overhead (O) shots. The analysis of the ending zone included the baseline and mid court zone (BMZ) (a shot that takes place behind the service line), the net zone (NZ) (a shot that takes place between the service line and the net) and the service zone (SZ) (only serves) (Fig 1). The analysis of the finishing shot by zone included the F played from baseline and mid court zone (F_BMZ), F played from the net zone (F_NZ), B played from the baseline and mid court zone (B_BMZ), B played from the net zone (B_NZ), FV played from the baseline and mid court zone (FV_BMZ), FV played from the net zone (FV_NZ), BV played from the baseline and mid court zone (BV_BMZ), BV played from the net zone (BV_NZ), O from the baseline and mid court zone (O_BMZ) and O played from the net zone (O_NZ).

**Table 1:** Observational instrument.

Criteria	Categories	Description
Player who hits	J1 J2 J3 J4	Player in the right of the winning pair Player in the left of the winning pair Player in the right of the losing pair Player in the left of the losing pair
Hitting place	G AB	Groundstroke (between the baseline and the service line) Attacking balls (between the service line and the net)
Type of shot I	F B	Forehand Backhand
Type of shot II	V O	Volley Overhead
Type of service	1 10 2 20 L	First service in First service out Second service in Second service out Let
Score	X-X	Game score (15, 30, 40, Ad, game)
Games	X-X	X takes values from 0 to 7
Set	X	1, 2 or 3

### Validity and reliability testing

The construct validity was obtained from the consistency of the conceptual framework by 6 tennis experts (certified coaches by the *Real Federación Española de Tenis*—Royal Spanish Tennis Federation- RFET), who reached 100% agreement for each of the categories. The validation was carried out using a virtual questionnaire, in which the experts could agree or disagree with the categorisation of each criterion. The reliability of the players who hit, hitting place, type of shots and type of service was determined after the observers had received a period of training in the application of the registration instrument and after reaching an agreement [30]. It was established by interobserver and intraobserver agreement on 200 actions in the game, with values of 0.888 and 0.985 respectively in the kappa coefficient. These values represent a very good reliability [31] (Table 2).

**Table 2:** Interobserver and intraobserver Kappa values of every criteria.

Criteria	Interobserver	Intraobserver
Player who hits	0.849	0.945
Hitting place	0.879	0.982
Type of shot 1	0.893	1.0
Type of shot 2	0.897	1.0
Type of service	0.926	1.0
**Mean**	**0.888**	**0.985**

### Statistical analyses

Statistical descriptors including mean and standard deviation were presented for relative frequencies. The Shapiro-Wilk test was used to verify normality of the variables. One-way ANOVA was used to compare differences among GS tournaments when variables were normally distributed. When significant differences were found, post-hoc tests using Bonferroni correction were performed and partial eta squared calculated effect sizes, small effect values were considered 0.01 –< 0.06, moderate effect values were considered 0.06 –< 0.14 and large effect values were considered ≥ 0.14. Data for percentage of F played from NZ, FRT played from BMZ, FV played from BMZ, B played from NZ, BV played from BMZ, and O played from BMZ showed non-normal distributions and were compared using Kruskal-Wallis and Mann-Whitney U tests. The level of significance was set at p < 0.05 for all analyses. Analyses were conducted using SPSS Statistics, version 26.0. The reliability was measured with the kappa coefficient [31].

## Results

### Type of finishing shot

**Fig 2:**
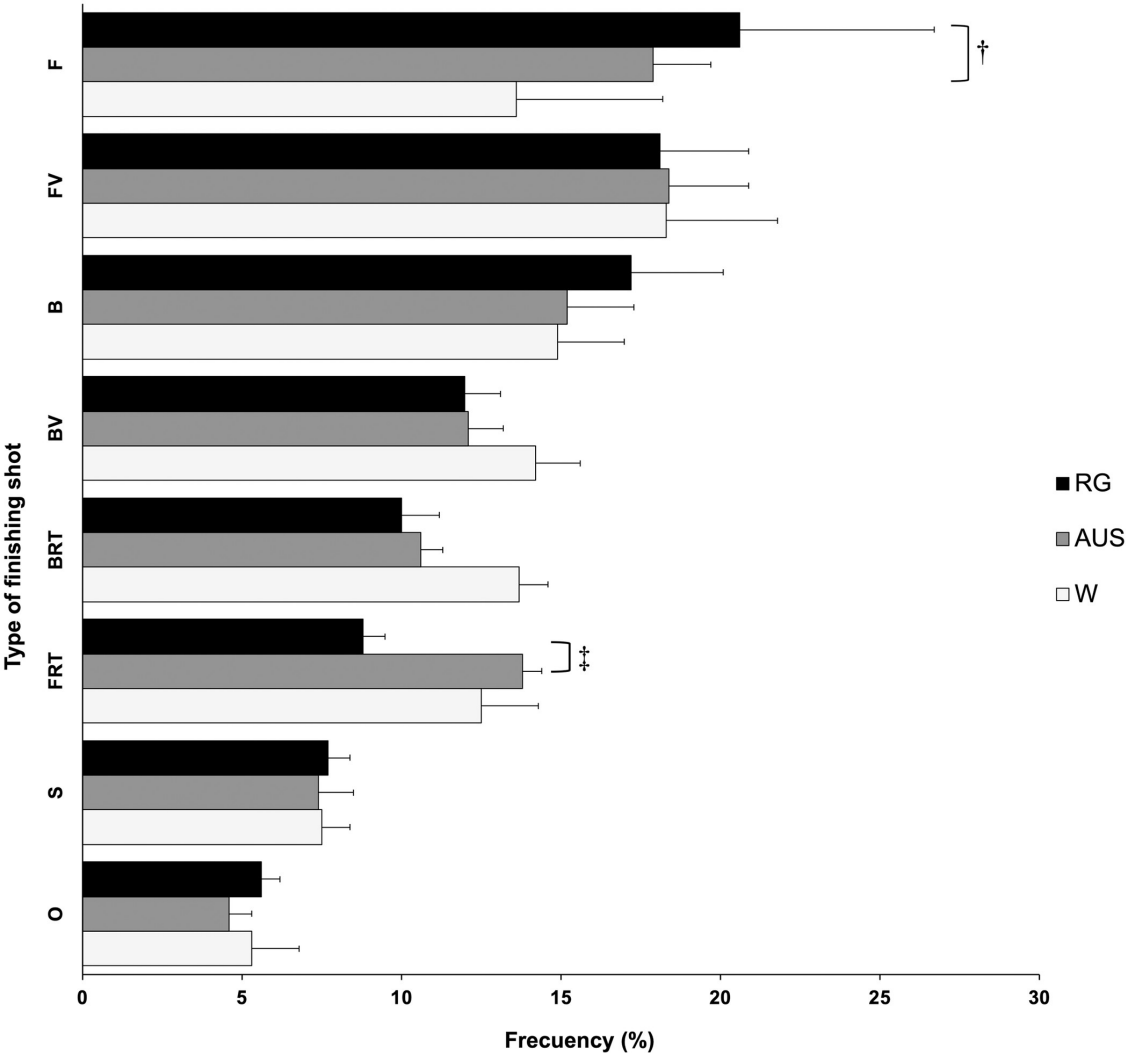
Finishing shots in the three Grand Slams. F, forehand; FV, forehand volley; B, backhand; BV, backhand volley; BRT, backhand return; FRT, forehand return; S, service; O, overhead; AUS, Australian Open; RG, Roland Garros; W, Wimbledon. † significant differences between RG and W; ‡ significant differences between AUS and RG.

Fig 2 shows the relative frequencies per match of the type of shots with which the points end in women’s doubles matches in the three GS tournaments. The most frequent shot was the FV (18.2%). Statistical significant differences were found for percentage of F (F = 4.566; p = 0.025; η^2^ = 0.337). Specifically, RG shows higher values than W in percentage of F (p = 0.023). Moreover, significant differences among surfaces (Kruskal-Wallis) were found in percentage of FRT (x^2^ = 6.38, df = 2, p = 0.041). Specifically, AUS shows higher values than RG in percentage of FRT (p = 0.026). No significant differences were identified among the different surfaces for the rest of type of finishing shots.

### Finishing zone

**Fig 3:**
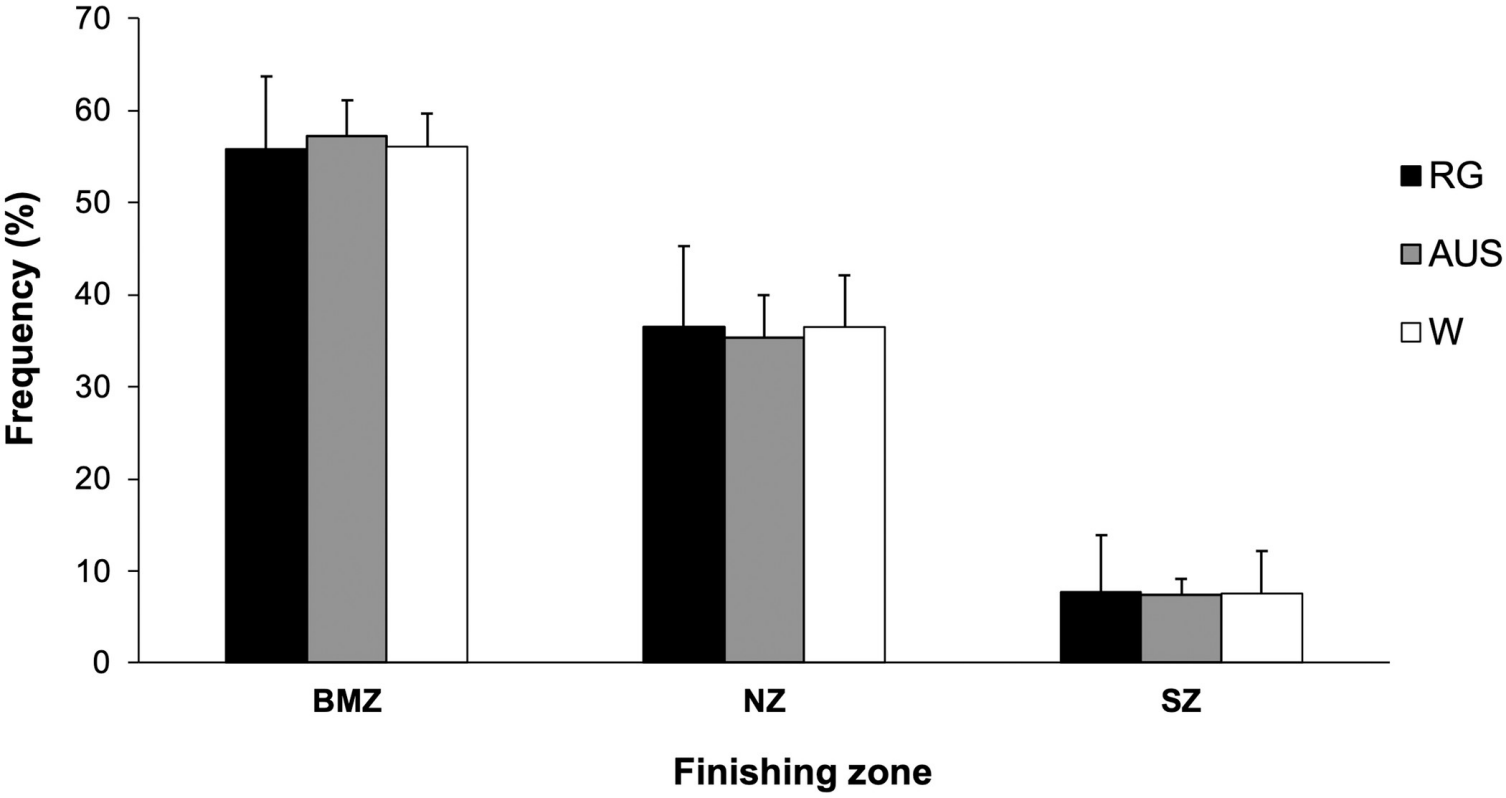
Mean percentage of the finishing zone of all the matches. NZ, net zone; BMZ, baseline and mid court zone; SZ, service zone; AUS, Australian Open; RG, Roland Garros; W, Wimbledon.

Fig 3 shows no significant differences among the different surfaces in the relative frequencies per match of the finishing zone with which the points end (Fig 3). On all surfaces, the most frequent area where the points end is BMZ (56.4%), followed by NZ (36.1%), while the least frequent is SZ (7.5%).

### Finishing shot by zone

**Fig 4:**
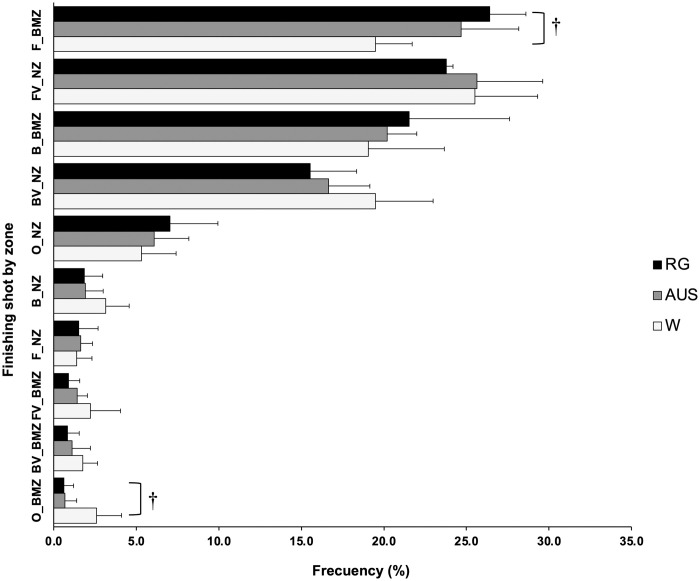
Finishing shots by zone in the three Grand Slams. F_BMZ, forehand baseline and mid court zone; FV_NZ, forehand volley net zone; B_ BMZ, backhand baseline and mid court zone; BV_NZ, backhand volley net zone; O_NZ, overhead net zone; B_NZ, backhand net zone; F_NZ, forehand net zone; FV_BMZ, forehand volley baseline and mid court zone; BV_BMZ, backhand volley baseline and mid court zone; O_BMZ, overhead baseline and mid court zone. † significant differences between RG and W.

Fig 4 shows the relative frequencies per match of the finishing shots by zone with which the points ends. Statistical significant differences were found for percentage of F_BMZ (F = 5.625; p = 0.013; η^2^ = 0.385). Concretely, RG shows higher values than W in percentage of F_BMZ (p = 0.011). Significant differences among surfaces (Kruskal-Wallis) were found in percentage of O_BMZ (x^2^ = 6.19, df = 2, p = 0.045), specifically, W shows higher values than RG (p = 0.038). No significant differences were identified among the different surfaces for the rest of finishing shots by zone.

## Discussion

Understanding the shots and court zones with which points end, provides valuable insights into professional women’s doubles tennis and can aid in structuring the practice accordingly. To the authors’ knowledge, this is the first study to describe the ending shot and ending zone of elite women’s doubles tennis on different playing surfaces of three different GS tournaments. Results showed the most frequent finishing shots were FV (18.2%), F (17.4%), B (15.8%), BV (12.8%), FRT (11.7%) and BRT (11.4%), while the most common ending zones were BMZ (56.4%) and NZ (36.1%). These findings highlight the importance in women’s doubles of mastering net play and efficient V and F to finish points decisively. With limited prior research examining point endings in doubles tennis, particularly women’s doubles, these results provide unique descriptive data to build from. Additionally, due to the limited research available on point endings, specifically in women’s doubles tennis, references to studies examining singles tennis will be made to provide additional context.

### Finishing shot

The results showed that the top women’s doubles tennis players finish most points with FV (18.2%), followed by F (17.4%), B (15.8%), BV (12.8%), FRT (11.7%) and BRT (11.4%). The finishing shot was defined as the last shot played regardless of its outcome (winner, unforced error, forced error). A study [6] in 2014 found that women’s doubles teams frequently use a classical formation, with the server’s partner standing at the net on the opposite side. This results in most volleys occurring near the net and on the sides, away from the center of the court [21]. Additionally, the doubles female players, especially using their first serve, attempt to move the returner by serving to the sides, even though they are not as effective in doing so as male players [20,32]. This leads to less aggressive returns, allowing the players volleying, to attack with their best shot, which could explain the percentage of FV point endings observed in the current study. Both women’s and men’s players try to move the receiver by serving to zones wide and T (area located in the middle of the court formed by the union of the service lines and the center line) with the first serve to take the initiative of the point, while with the second serve both take lower risks and mainly serve to zone body (to the center lane of the service box). The effectiveness of the first serve in men’s doubles match is higher in all the directions inside the service box (i.e. wide, T and body), while there are only differences in the effectiveness of the second serve in the zone body of the advantage service box. In general, the effectiveness of the serve in women’s matches may not have the same efficacy of that found in the men’s matches, but does show a similar tactical approach [20].

If we take in account the most frequent shots in women’s doubles, we find the F in first place (26%) [22], but it’s the second shot with which points finish the most (17.4%). Thus, the percentage of F shots during rallies is likely higher than the percentage of points ending with F. Additionally, more points are finished with F in RG than in W, but the F is hit more frequently at W. This discrepancy is likely because the slower clay courts allow more time for players to get in position and execute the F shot better. Overall, while the F is a common rally shot for women, other shots like the volley are more frequent point-enders.

The B is the third most common shot to finish points at 15.8% and is the second most frequent rally shot at around 25% in women’s doubles [22]. Similar to F, fewer points end with backhands compared to the frequency of B shots during rallies. This seems to indicate the lower offensive potential and effectiveness of the B compared to the F in women’s doubles. Players seem to prefer hitting F when possible and utilize the B more during rallies rather than to finish points. Around 16% of shots in women’s doubles are V [22]. However, taking into account FV and BV, approximately 31% of points end with a V. Despite V being the fourth most frequent shot [22], it is used to finish points more than any other shot, except the F and B together. This is likely because V are more definitive than other shots, frequently ending the point when hit. This shot, apart from being more definitive, is much more frequent in doubles than in singles tennis [22,33]. Comparing FV and BV, around 8.6% of shots in women’s doubles are FV and 7.6% are BV [22]. The order is the same for finishing shots, with 18.2% of points ending with FV and 12.8% with BV. Though no significant differences were found, the FV percentage is slightly higher on hard courts and clay, while the BV percentage is slightly higher on grass [22]. However, similar to rally shots, more points end with FV than BV on all three surfaces. Furthermore, although no significant differences were found, it is interesting that there are more FV played from NZ in RG than in W, but more BVs played from NZ in W than in RG. This finding suggests that playing on slower surfaces, such as RG, may offer players more time at the net to play FV.

The S is the second most frequent shot at around 25% when including first and second S [22]. However, only 7.5% of points end with a S. This aligns with findings of previous studies that have shown a lower effectiveness of S in women’s doubles compared to men’s doubles [20]. Additionally, if the S does not immediately end the point, playing a V will likely finish it instead, as it is the most common ending shot. In this study, return errors did not count as S points. Finally, regarding O, they are only used by players around 2% of the time [21,22]. However, 5.2% of points end with O, probably because it is the most definitive shot and when it’s used there are few possibilities of returning the ball. The finishing shot values for FRT (11.7%) and BRT (11.4%) are similar. This is likely because modern S variety provides fewer repeated serve locations, especially with first S [20]. Thus, both FRT and BRT are utilized. The high frequency of V highlights how players are actively involved at the net, leading to more V rallies and points ending with V as the most definitive shot along with O. F is the second most common ending shot given that over 25% of shots in women’s doubles are F. Consequently, many forced errors, unforced errors, and winners occur from this shot. In summary, V finish points most often due to being offensive and close to the net play. However, F and B remain the most common ending shots given their prevalence during baseline rallies.

### Ending zone

The results showed that approximately 56.4% of points ended in the BMZ, while 36.1% ended in the NZ and just 7.5% ended in the SZ. This aligns with previous research indicating around 50% of shots in doubles come from the BMZ, 18% from the NZ, and the remainder from serves in the SZ [22]. The higher frequency of net play compared to singles is likely because there is always two of the four players at the net for the beginning of each point [22,33]. Given the prevalence of net play, it’s logical that over one third of points conclude in the NZ with aggressive V, the second most common ending shot per the current study’s results. Specifically, FV ended 18.2% of points, followed by BV at 12.8%. 33.2% ended with groundstrokes from the baseline, including 17.4% with F and 15.8% with B. This further highlights the importance of net play, V, and F in finishing points decisively in professional women’s doubles.

## Limitations and prospects for future studies

The study’s methodology, while robust, is limited by its observational design, as it focuses exclusively on high-level women’s doubles matches at GS touraments. This constraint may not capture the full diversity of strategies and outcomes present at different competitive levels. Future studies should consider analyzing matches from a broader range of tournaments and levels to provide a more comprehensive understanding of the dynamics in women’s doubles tennis. Another significant limitation is the lack of consideration for player limb dominance, which could influence the effectiveness of certain shots and the set up of the players. Incorporating limb dominance and the relationship between shot speed and finishing shots into future research could offer deeper insights into match play dynamics.

## Practical applications

From a tactical standpoint, this study highlights the importance of net play and the strategic selection of finishing shots in women’s doubles tennis. Coaches and players should focus on developing skills that enable aggressive net play, such as effective volleying and overhead shots, to dominate the net zone. Emphasizing the practice of specific shots that were found to be more prevalent in successful point endings, like the forehand volley and forehand from the baseline and mid-court zone, could be beneficial. Tailoring training to simulate match conditions across different surfaces can also prepare players to adapt their game strategically to the unique demands of each Grand Slam surface, enhancing their competitiveness at the highest level of the sport.

## Conclusions

In women’s doubles tennis the most frequent finishing shots were the F and B together, followed by the V, FRT, BRT, S and O, while the most common ending zones were BMZ and NZ. Despite volleys not being used as often when compared to groundstrokes over the entire match, volleys are more likely to end points in women’s double’s tennis. These results suggest that in women’s doubles tennis, controlling the game in the net area, as well as being efficient with V and F shots, is very important and can be decisive in the outcome of the match. Consequently, this study may be useful for coaches from both a tactical and technical standpoint when designing practices for professional doubles women’s players.

## Supporting information

S1 File Codebook. (XLSX)

S2 File Dataset. (XLSX)
